# Wound of the Stomach with Protrusion

**Published:** 1852-04

**Authors:** W. Ashby

**Affiliations:** Culpeper, C. H., U. S.


### RECORD OF MEDICAL SCIENCE.

### SURGERY.

*Wound of the Stomach with Protrusion.*—By W. Ashby, M. D., of Culpeper, C. H., U. S.—A negro boy, aged 6, the property of Mr. R. B., fell upon a pair of sheep-shears, which he had in his hand, whilst running down a hill. The instrument penetrated the stomach obliquely from above, just grazing the left side of the sternum and edges of the ribs, making a flaplike orifice in the integuments. I was called in consultation by my friend, Dr. P. C. Slaughter, and found nearly the whole stomach protruded, and discharging its contents through an aperture about three-quarters of an inch in length. Aware of the controversy which has so long existed among able surgeons, on either side, as to the propriety of stiching the stomach or bowels, the everted edges and gaping appearance of the wound in the stomach made it necessary, I thought, that a stitch should be taken. To avoid irritation, as much as possible, with the finest needle and silk I ventured to take a single stitch through the middle of the wound. Before I saw the case, Dr. S. had made some efforts to restore the organ to its natural position, but it did not occur to me at the time that I should have any serious difficulty in replacing it, at least after enlarging the orifice a little. But such was the unruly nature of the boy—his violent screaming and resistance, the nausea and vomiting which constantly attended the handling of the stomach—that notwithstanding I enlarged the orifice several times to a considerable extent, our best efforts not only failed to restore the organ, but it seemed to protrude the more. At this juncture, fearing the irritation resulting from further efforts, I suggested the use of chloroform, notwithstanding the necessary delay of having to send several miles for it. Whilst under its influence, I found it necessary again to enlarge the aperture slightly, and then had no farther difficulty, although the boy vomited as freely as before from handling the organ. The wound of the integument was rather ragged in its appearance, and of course a little bruised by our efforts. The wound of the stomach was brought directly opposite the tegumentary wound, and gently retained within its verge. A single stitch, patent lint, with cold water and a bandage completed the dressing. The patient was placed on his side, absolute rest enjoined, and soon afterwards a large dose of opium was administered. From the time of the accident until the completion of the dressing six hours intervened, and yet the boy retained his strength most remarkably. Under the influence of the opium our patient rested well the first night.

2d day. This morning the pulse is a little excited, and face flushed. V. S. to make a decided impression; and this was repeated twice during the day, and opium after each bleeding—absolute diet enjoined—but the boy desires no food.

3d. The wound had a healthy appearance, but tenderness of the abdomen and tympanitis greatly increased our fears as to the result. The pulse feeble and quick; the bowels not moved since the accident. Turpentine enema and a succession of blisters were ordered, and after the bowels were moved the opium was resumed.

4th. Our patient evidently improved, tympanitis and tenderness diminished, pulse more quiet, countenance and general aspect of things more encouraging; takes a little hot water tea this morning for the first time; gum water and opium continued.

5th. The wound not healed by the first intention; has a dark spot immediately over the wound of the stomach, and is discharging a very offensive sanious matter. A soft poultice, and the same prescription continued.

6th. The ligature came out this morning. The same prescription continued. From this date the boy gradually recovered, without any particular change in the treatment.

*Remarks.*—1st. It has occurred to me, that possibly it would have been better for me to have restored the stomach, at least partially, before the stitch was taken, as I ran the risk of breaking out the ligature by the subsequent efforts at reduction; and I am sure that the accumulation of gas, though some escaped with an audible sound several times, did not increase the difficulty.

2d. This case was admirably adapted to the use of chloroform, and illustrates most happily its incalculable value when used with discrimination.

3d. As your journal is eminently practical in its character, for the benefit of the younger members of the profession, it may not be amiss to allude briefly to what I conceive to be a most important principle in our profession, viz., that an *inflamed or diseased organ must have rest.* In this case, the stomach instinctively sensible of its wounded and disabled condition, refused most emphatically, for four days, to receive any nourishment—not even gum water—and but very little of anything for about ten days, notwithstanding the entreaties of master and friends, contrary to our orders.

An inflamed eye instinctively excludes the light from itself, so that the physician who interrogates Nature intelligently, at once gets the idea of confining his patient to a dark room, and thus putting the organ entirely to rest. When the lungs are inflamed the patient breathes as much as possible by the abdominal muscles, and lymph is thrown out, gluing the organ to the side, doubtless to prevent motion and friction as much as possible. The same thing is true of inflamed bowels; and because some constipation, the result of this principle exists, I have known great error—and I may say even death—to result from goading and stimulating the organ with drastic purgatives. This principle of rest is susceptible of very extensive application in practice; and any inflammation can be cured, I believe, to which it can be applied. The immortal Physic, always true to the laws of Nature, recognized this principle in the treatment of coxalgia and other diseases of the joints. In conformity to this important law of the animal economy, in the above case, we gave opium freely, to prevent nervous and vascular reaction; and by thus aiding in keeping the wounded orgen in a profound state of repose, it contributed, it is believed, no little to the favorable result.—*Stethoscope, (Virginia,) United States.*

